# Predicting the Potential Geographical Distribution of *Scolytus scolytus* in China Using a Biomod2-Based Ensemble Model

**DOI:** 10.3390/insects16070742

**Published:** 2025-07-21

**Authors:** Wei Yu, Dongrui Sun, Jiayi Ma, Xinyuan Gao, Yu Fang, Huidong Pan, Huiru Wang, Juan Shi

**Affiliations:** 1 School of Science, Beijing Forestry University, Beijing 100083, China; 2 College of Forestry, Beijing Forestry University, Beijing 100083, China; 3 Sino-France Joint Laboratory for Invasive Forest Pests in Eurasia, Beijing Forestry University, Beijing 100083, China

**Keywords:** *Scolytus scolytus*, Dutch elm disease, species distribution model (SDM), biomod2, potential geographical distribution, climate change, ecological niche modeling, pest risk analysis

## Abstract

Dutch elm disease is one of the most devastating plant diseases, primarily spread through bark beetles. *Scolytus scolytus* is a key vector of this disease. In this study, distribution data of *S. scolytus* were collected and filtered. Combined with environmental and climatic variables, an ensemble model was developed using the Biomod2 platform to predict its potential geographical distribution in China. The selection of climate variables was critical for accurate prediction. Eight bioclimatic factors with high importance were selected from 19 candidate variables. Among these, the three most important factors are the minimum temperature of the coldest month (bio6), precipitation seasonality (bio15), and precipitation in the driest quarter (bio17). Under current climate conditions, suitable habitats for *S. scolytus* are mainly located in the temperate regions between 30° and 60° N latitude. These include parts of Europe, East Asia, eastern and northwestern North America, and southern and northeastern South America. In China, the low-suitability area was estimated at 37,883.39 km^2^, and the medium-suitability area at 251.14 km^2^. No high-suitability regions were identified. However, low-suitability zones were widespread across multiple provinces. Under future climate scenarios, low-suitability areas are still projected across China. Medium-suitability areas are expected to increase under SSP370 and SSP585, particularly along the eastern coastal regions, peaking between 2041 and 2060. High-suitability zones may also emerge under these two scenarios, again concentrated in coastal areas. These findings provide a theoretical basis for entry quarantine measures and early warning systems aimed at controlling the spread of *S. scolytus* in China.

## Simple Summary

The bark beetle *Scolytus scolytus*, a major vector of Dutch elm disease, threatens elm trees globally. Our study employed advanced ecological modeling to assess its potential distribution across China under current and future climate conditions. The results reveal that while most of China currently offers only marginally suitable habitats (37,883 km^2^), concentrated primarily in eastern coastal areas, climate change may significantly expand these ranges. By the mid-21st century, moderate-suitability zones could grow substantially, particularly under high-emission scenarios, with new risk areas emerging in central and southern provinces. These projections emphasize the growing invasion risk and underscore the urgency for strengthened border biosecurity, targeted surveillance in vulnerable regions, and proactive management strategies to safeguard China’s ecologically and economically important elm resources.

## 1. Introduction

Biological invasion is recognized as one of the five major global environmental challenges of the 21st century. It poses serious threats to ecological stability, economic development, and public health, particularly endangering biodiversity [1,2]. With the rapid expansion of international trade and tourism, the frequency and severity of biological invasions have increased significantly in China. This growing threat demands urgent attention. Consequently, the development and refinement of ecological risk assessment systems for invasive alien species have become a critical and pressing task [3].

*Ulmus* spp. mainly distributed in temperate regions of the Northern Hemisphere [4]. Owing to its strong adaptability, excellent resistance, and an esthetically pleasing tree form, it is extensively applied to afforestation, industrial production, and landscapes [5]. Furthermore, elm trees possess medicinal properties and serve as a significant forest resource [6]. Dutch elm disease (DED) is a devastating forest disease that has historically been widespread across Europe, North America, and Central Asia, exerting a large impact on local elm resources [7]. For instance, *U. minor* as a key component of European riparian forests was on the verge of extinction due to DED [8]. This disease is vectored by bark beetles of the genera *Scolytus* and *Hylurgopinus*, with long-distance propagation related to human activities [9]. The dispersal dynamics of DED and its vectors continue to receive attention from quarantine departments of various countries.

*Scolytus scolytus* Fabricius (1775) (Curculionidae: Scolytinae), known as an important carrier of DED pathogens, is currently distributed in various European countries, including Sweden, France, and Russia [10]. An economic assessment evaluated the damage caused by invasive pests and pathogens in elm plantations in St. Petersburg, Russia [11]. The study revealed that Dutch elm disease, driven by *S. scolytus*, *S. multistriatus*, and other elm bark beetles in combination with DED pathogens, led to the death of at least 37,000 elm trees between 2001 and 2020, resulting in economic losses exceeding 50 billion rubles. Due to its key role in the transmission of DED and its considerable invasive potential, *S. scolytus* has been categorized as an A1 or A2 quarantine pest by several countries and international organizations, including Chile, Argentina, and the Asia and Pacific Plant Protection Commission (APPPC) [12].

Climate change has altered the abundance and distribution of species, and is even associated with species extinction [13]. Species distribution models (SDMs) typically integrate the presence (or absence) of species with environmental covariates to predict their potential distribution or ecological preferences [14]. Common SDMs encompass a variety of types, such as MaxEnt, CLIMEX, GLM, and mechanistic niche modeling based on biophysical ecology [15,16]. Currently, they have been widely utilized in many research fields, including evaluating the impact of climate and land changes on species, invasive risk assessment, and conservation natural reserves [17]. To reduce the prediction error of a single model, ensemble models combine multiple models though weighted averaging and other methods to enhance predictive performance [18,19]. Zhao et al. applied Biomod2 to predict the potential distribution of *Phoracantha semipunctata* under climate change [20]. Their research indicated that the predictive results of EM were reliable and provided references for the preventive measures of *P. semipunctata*. Similarly, another research analyzed the distribution pattern of Chinese *Ziziphus jujuba* through optimized Biomod2 and MaxEnt [21]. The prediction accuracy was greatly increased by integrating the model and screening for environmental variables. This study not only supported the conservation and sustainable utilization of jujube resources, but also demonstrated the versatility of Biomod2 in plant distribution modeling. In summary, ensemble models (especially based on Biomod2) have become popular tools in related research.

*S. scolytus* has not been recorded in China to date. However, elm trees with a long-cultivated history are widely distributed in China, and various areas have close trade relations with European regions. Consequently, the cross-border spread of *S. scolytus* possesses both anthropogenic and natural conditions. Although China has established quarantine and identification standards for *S. scolytus*, pest risk analysis is still lacking. There has also been no research about the potential global and Chinese distribution of this pest, while the theoretical system for early detection and warning remains imperfect.

To mitigate the potential risk of *S. scolytus* being introduced into China and causing harm, this study established an ensemble model by Biomod2 to predict the geographical distribution of this pest under near-current climatic conditions. We identified the significant environmental variables and further evaluated the changes in the suitable areas for *S. scolytus* in China under different future climate scenarios.

## 2. Materials and Methods

### 2.1. Environmental and Occurrence Data Collection

Nineteen bioclimatic variables known to influence species distribution were selected as environmental predictors. Current climate data were obtained from the WorldClim database (version 2.1; http://www.worldclim.org (accessed on 14 October 2024)) at a spatial resolution of 2.5 arc-minutes, representing the baseline period from 1970 to 2000. Future climate projections were also sourced from WorldClim, using the BCC-CSM2-MR global climate model. The following four SSP scenarios were considered: SSP126, SSP245, SSP370, and SSP585. These projections cover the period 2021–2100 and are divided into four intervals as follows: 2021–2040, 2041–2060, 2061–2080, and 2081–2100.

**Figure 1:**
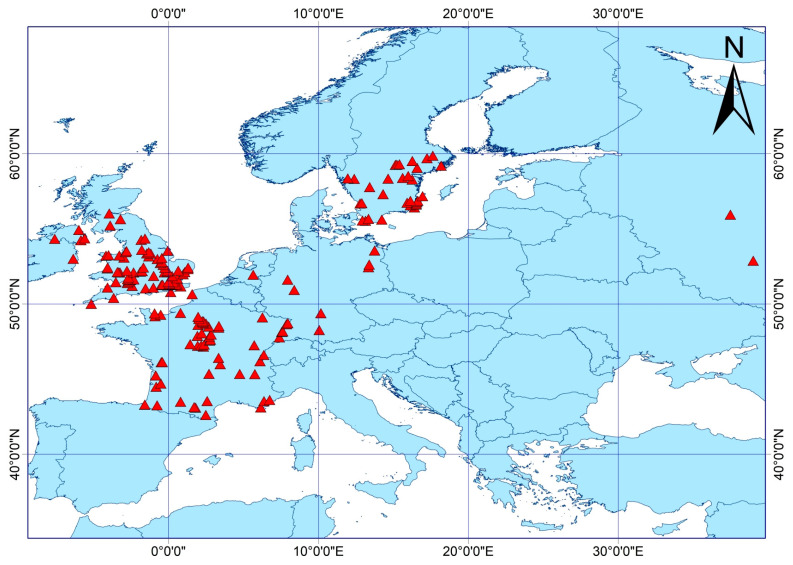
Occurrence records of *S. scolytus*.

Species occurrence records for *S. scolytus* were downloaded from the Global Biodiversity Information Facility (GBIF; https://doi.org/10.15468/dl.8zuw3y (accessed on 14 May 2024)) [22]. The data were initially cleaned in Microsoft Excel by removing duplicates, erroneous entries, and spatially inconsistent records. Under the selected spatial resolution, each climate grid covers approximately 21.0 km^2^. To reduce spatial autocorrelation, a buffer analysis was conducted in ArcGIS 10.8, using a 2.5 km radius to ensure that only one occurrence point was retained per grid cell. After filtering, a total of 173 unique occurrence points were retained and saved in “.CSV” format. The spatial distribution of these records is shown in Figure 1, where red triangles indicate the documented locations of *S. scolytus*.

### 2.2. Parameter Setting and Model Ensemble

To reduce the uncertainty associated with single-algorithm predictions, nine species distribution modeling algorithms were selected and implemented using the Biomod2 platform [23,24,25,26]. These included GLM, GAM, CTA, ANN, SRE, FDA, MARS, RF, and MaxEnt [27,28] (Table 1). Among them, MaxEnt has been widely applied in species distribution modeling [28,29]. Its integration within Biomod2 allows for both efficient performance and seamless comparison with other algorithms, thereby enhancing the robustness and accuracy of ensemble predictions.

For each algorithm, we generated 80 random pseudo-absence records within the global background, excluding Antarctica, for model calibration. Each model was trained using 80% of the presence and pseudo-absence data. Model performance was evaluated using the AUC and the TSS value on the remaining 20% of the dataset. TSS scores were calculated for each algorithm, and only those with TSS > 0.8 were incorporated into the final ensemble model. The potential geographic distribution of *S. scolytus* in China was simulated using the ensemble model. After optimization and adjustment, the final prediction results were projected and exported in .tif format.

#### 2.2.1. Environmental Variable Selection

To reduce multicollinearity and autocorrelation among environmental predictors, a systematic screening of variables was performed. An excessive inclusion of correlated variables may lead to model overfitting and reduce predictive accuracy. Therefore, Spearman’s rank correlation coefficients were calculated for all 19 bioclimatic variables. When the correlation coefficient between two variables exceeded 0.75, the variable with the higher contribution to the model performance was retained, while the other was excluded [38]. The final set of selected variables is described in detail in Section 3.2.

#### 2.2.2. Classification and Threshold Definition of Habitat Suitability

The output from the optimized ensemble model was imported into ArcGIS 10.8 for reclassification and visualization. In the output layer, the predicted value (*p*) represents habitat suitability, ranging from 0 to 1. A higher value indicates greater environmental suitability for the species. To facilitate interpretation, the suitability levels were classified based on predicted values (*p*), and the study area was divided into four suitability classes as follows: highly suitable (0.7 ≤ *p* < 1.0), moderately suitable (0.5 ≤ *p* < 0.7), slightly suitable (0.3 ≤ *p* < 0.5), and unsuitable (*p* < 0.3) [39].

#### 2.2.3. Model Performance Evaluation and Variable Importance

The ROC curve and TSS were used to evaluate model performance [40,41]. The ROC curve reflects the model’s sensitivity and specificity by plotting the TPR against the FPR under varying thresholds. The AUC value quantifies the model’s overall ability to distinguish between presence and absence records. In contrast, TSS is threshold-dependent and combines sensitivity (TPR) and specificity (1-FPR) to assess classification accuracy at a specific cutoff. A higher AUC indicates better overall discriminatory power, while a higher TSS reflects improved classification ability under a defined threshold. Together, AUC and TSS provide a comprehensive assessment of predictive performance across all candidate models.

The relative importance of environmental variables was calculated using the Biomod2 package. This was performed to evaluate the influence of each predictor on model output. Specifically, univariate model predictions were compared with the original data to determine the contribution of individual variables to habitat suitability. Importance values were calculated as 1 minus the correlation coefficient between the observed values and the predicted results of each univariate simulation. Higher values indicate a stronger influence on the species predicted distribution. Conversely, a value of zero suggests no significant contribution to habitat suitability.

## 3. Results

### 3.1. Evaluation of Model Accuracy

**Figure 2:**
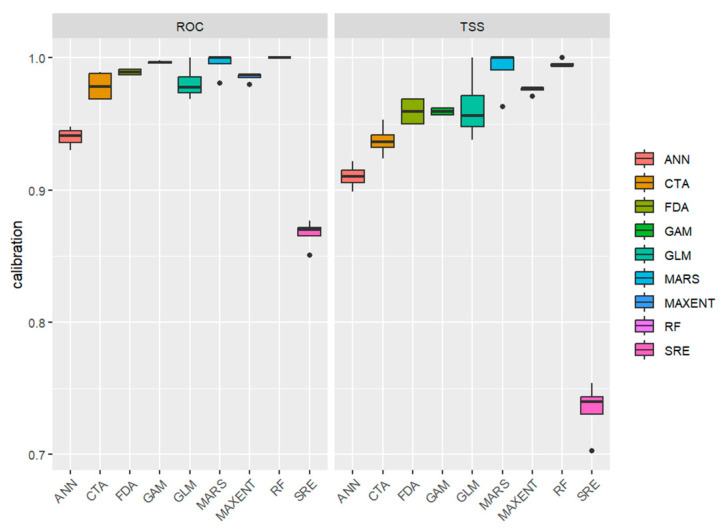
ROC and TSS box plot of the model.

In this study, the use of the Biomod2 packages for model ensemble methods demonstrated notable advantages. Multiple models achieved excellent performance on two key metrics: ROC and TSS, with FDA, MARS, MAXENT, and RF models reaching average values approaching 0.99 (Figure 2). Through ensemble methods, we significantly enhanced model generalization by leveraging the strengths of multiple models.

### 3.2. Screening of Environmental Factors

**Figure 3:**
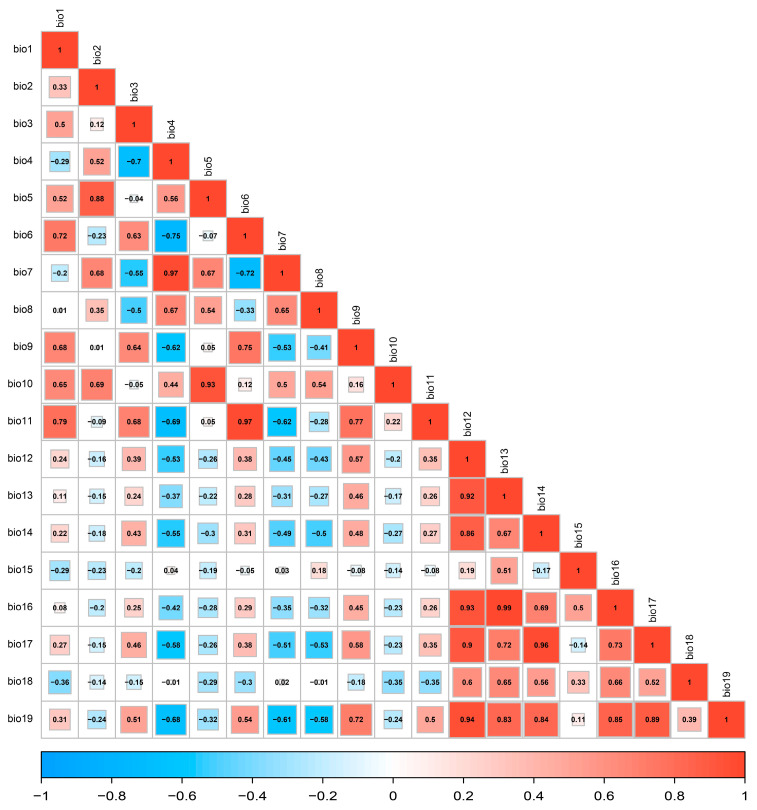
Correlation matrix of environmental factors.

In the preliminary variable screening, we conducted a correlation analysis utilizing the Spearman correlation coefficient for 19 candidate environmental factors (Figure 3). Ultimately, eight environmental factors were retained for modeling purposes.

Based on this, the average importance values of the eight environmental factors in the model were further calculated, along with their proportions in total importance (Table 2). Among these factors, the minimum temperature of the coldest month (bio6) contributed the most, accounting for 35.7%. This was followed by the precipitation variation (bio15) and the precipitation of the wettest season (bio17). These findings indicate that fluctuations in temperature and precipitation are key factors influencing the distribution of suitable habitats for this species.

### 3.3. Global Potential Geographic Distribution of S. scolytus Under Current Climatic Conditions

The global potential habitats suitable for *S. scolytus* are primarily concentrated in temperate and some subtropical regions. The extent of highly suitable areas is considerable, predominantly located in Europe, eastern North America, southern South America, southeastern Oceania, and parts of Asia. Theoretically, Europe presents the largest expanse of highly suitable habitats worldwide, encompassing nearly the entirety of Western, Central, and Eastern Europe, particularly within temperate zones. In eastern North America, specifically in the northeastern United States and the Great Lakes region, the area of highly suitable areas is also substantial due to favorable environmental conditions. In South America, these habitats are primarily found in the temperate maritime climate zones of southern Chile and southern Argentina, while in Oceania, they comprise the southeastern coast of Australia and Tasmania. Certain regions in Asia, such as parts of Japan and the coastal areas of the Russian Far East, also offer suitable environmental conditions for the proliferation of *S. scolytus*. Moderately suitable and slightly suitable areas are mainly distributed in the transitional zones from subtropical to temperate regions, including southeastern China, the central plains of the United States, the low- and medium-altitude regions of the Andes, and parts of southern Africa. The distribution of suitable areas exhibits significant latitudinal characteristics, predominantly concentrated in the temperate zones between 30° N and 60° N, with particularly notable suitable areas in coastal regions.

**Figure 4:**
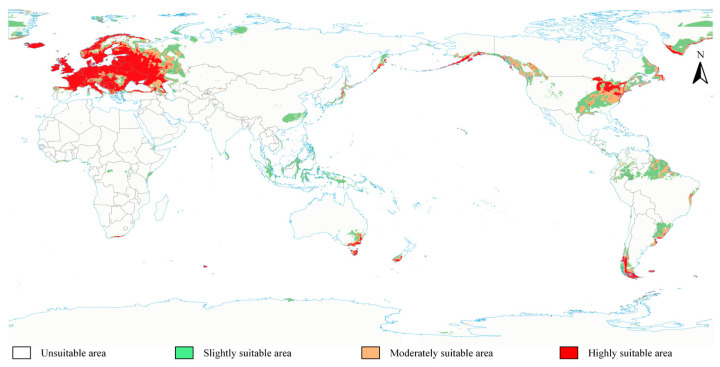
Distribution of the *S. scolytus* in the suitable habitat area under the current climate.

The prediction results, as illustrated in Figure 4, indicate that the global potential suitable habitats for *S. scolytus* are predominantly distributed in temperate zones between 30° and 60° north latitude. These habitats include regions in Europe, eastern Asia, eastern and northwestern North America, and southern and northeastern South America. Among these, Europe has the highest concentration of highly suitable areas, covering almost the entirety of Western, Central, and Eastern Europe. Additionally, highly suitable areas are also present in southeastern Australia, the Great Lakes region of the United States, the coastal areas of the Russian Far East, southern Chile, and southern Argentina. These regions encompass various climate types, including temperate maritime and Mediterranean climates. Moderately suitable and slightly suitable areas are concentrated in transitional areas from subtropical to temperate zones, such as southeastern China, the central plains of the United States, mid- to low-altitude regions of the Andes, and parts of southern Africa. The distribution of suitable zones exhibits significant latitudinal characteristics, with particularly prominent highly suitable coastal areas.

### 3.4. Potential Geographic Distribution of S. scolytus in China Under Current Climatic Conditions

**Figure 5:**
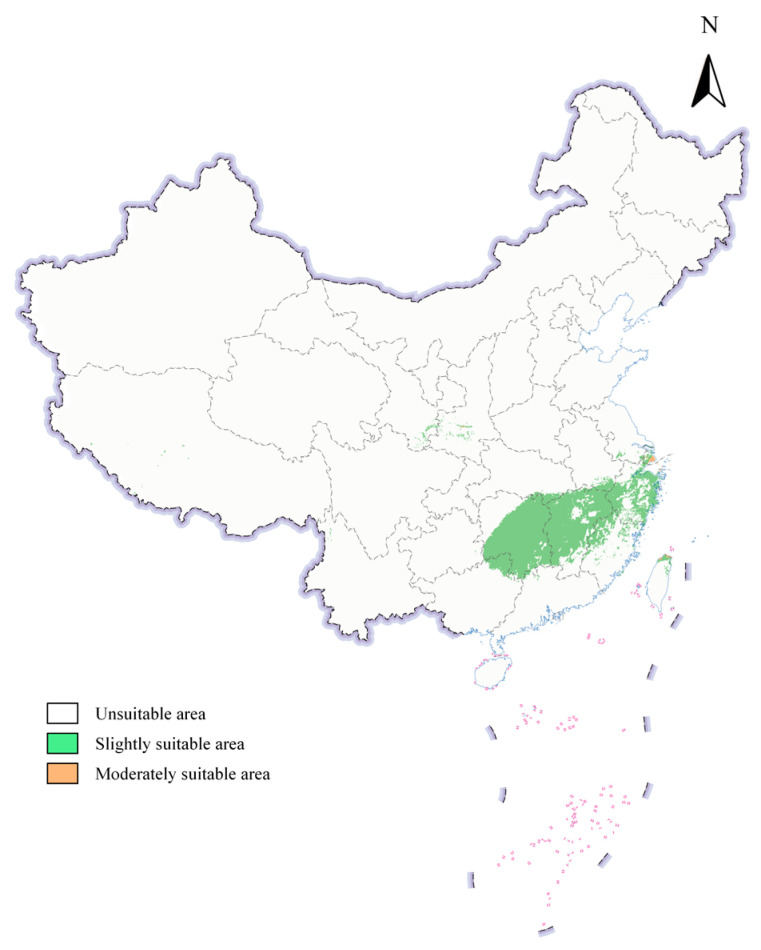
Distribution of the *S. scolytus* in China under the current climate.

Under the current climatic scenario (Figure 5), the distribution of suitable areas for *S. scolytus* in China is predominantly characterized by slightly suitable areas, which cover an area of 37,883.39 km^2^. In contrast, the medium-suitability zones are significantly smaller, encompassing only 251.14 km^2^, with no high-suitability zones identified. The slightly suitable areas are extensively distributed across provinces including Zhejiang, Shanghai, Jiangsu, Fujian, Anhui, Jiangxi, Hunan, Guangxi, Taiwan, Gansu, and Shaanxi. Conversely, the moderately suitable areas are limited in area and more concentrated, primarily located in the coastal regions of Shanghai. This distribution pattern indicates that the potential suitable areas for *S. scolytus* in China are predominantly slightly suitable areas, while the moderately suitable regions remain relatively confined.

### 3.5. Potential Geographic Distribution of S. scolytus in China Under Future Climate Conditions

Under future climate scenarios (SSP126, SSP245, SSP370, and SSP585), the potential suitable habitats for *S. scolytus* in China are projected to remain dominated by low-suitability zones, which account for the vast majority of the total suitable area. Notably, under SSP370 and SSP585 scenarios, these low-suitability zones exhibit extensive coverage across nearly all provinces nationwide. Medium-suitability areas show increased concentration under SSP370 and SSP585, peaking during 2041–2060, suggesting enhanced climatic favorability for the species under these scenarios. High-suitability zones remain extremely limited, appearing sporadically only under SSP370 and SSP585, suggesting that a limited number of highly suitable habitats may exist under these specific scenarios. Overall, intensified climate change (particularly under SSP370 and SSP585) may expand the suitable habitats of *S. scolytus* in China, with medium-suitability areas demonstrating the most significant spatial growth.

**Figure 6:**
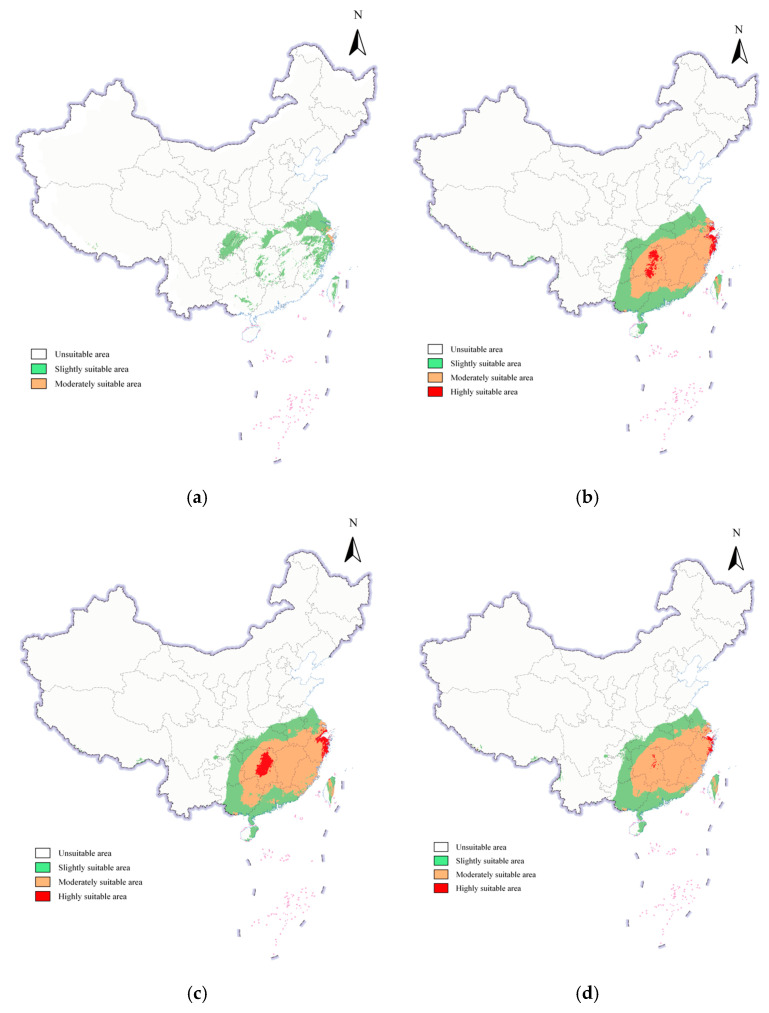
Potential geographic distribution of *S. scolytus* in China under future climate scenarios. (**a**) SSP370 scenario (2021–2040); (**b**) SSP370 scenario (2041–2060); (**c**) SSP585 scenario (2021–2040); (**d**) SSP585 scenario (2041–2060).

Under the SSP370 scenario, projections for 2021–2040 indicate low-adaptation zones of *S. scolytus* in China reached 41,349.09 km^2^ (a 9.15% increase from current conditions), expanding into new provinces including Chongqing and Sichuan (Figure 6a). Moderately suitable areas expanded to 914.28 km^2^ (a 3.6-fold increase compared to the current climate scenario), primarily concentrated in coastal regions of Zhejiang and Jiangsu provinces, excluding Shanghai. By 2041–2060 (Figure 6b), suitable regions showed marked growth and spatial concentration in central and southern China, marked by the emergence of larger high-adaptation zones. Quantitatively, slightly suitable areas totaled 56,974.90 km^2^, moderately suitable areas surged to 67,370.18 km^2^, and highly suitable areas reached 5826.35 km^2^. Moderately suitable areas spanned across Jiangsu, Shanghai, Zhejiang, Fujian, Anhui, Jiangxi, Hunan, Hubei, Guangdong, and Guangxi. Notably, regions with established moderately suitable clusters during 2021–2040 (Zhejiang, Shanghai, Jiangsu) developed highly suitable clusters by 2041–2060, while Hunan Province showed a substantial growth in high-adaptation coverage. This progression demonstrates that climate intensification has broadened the adaptive range of *S. scolytus* in China, particularly positioning central and southern regions as emerging high-adaptation hotspots.

Under the SSP585 scenario (Figure 6c,d), the spatial distribution of suitability areas from 2021 to 2040 mirrors the SSP370 pattern projected for 2041–2060, featuring extensive moderately and highly suitable areas. These priority habitats cluster predominantly in southern China, focusing on coastal and central provinces. Consistent with the SSP370 scenario, highly suitable areas are concentrated in Shanghai, Jiangsu, Zhejiang, and Hunan. Analysis of temporal shifts reveals that by 2041–2060, slightly and moderately suitable areas expand in coverage, while high-suitability areas contract significantly. Nevertheless, these high-suitability hotspots persist in Zhejiang, Shanghai, Jiangsu, and Hunan. This trend suggests elevated invasion risks for southern China under SSP585, demanding intensified monitoring and preemptive controls to mitigate ecological threats.

## 4. Discussion

### 4.1. Methodological Discussion

In this study, the biomod2 package was used to construct an ensemble model by integrating multiple algorithms. This approach minimized randomness and bias often associated with single-model predictions. Each individual model achieved high accuracy, while the ensemble model showed particularly robust performance. The composite model can capture patterns and relationships in the data more comprehensively, yielding improved predictions for new data. Boxplot analysis indicates that the median approaches 1 with tightly clustered data distribution, indicating strong alignment between predictions and the actual situation, confirming the model’s accuracy and reliability (Figure 1). These findings establish a robust scientific foundation for decision-making, strengthening confidence in the predictive outcomes.

However, only 19 environmental variables were considered. The potential effects of additional factors on the geographical distribution of *S. scolytus* were not included. Moreover, climate change impacts on the life history of the pathogen-vector-host system were not incorporated into the modeling framework [42]. Future studies could integrate other biological elements (e.g., host plant distribution, interspecific competition), abiotic drivers (e.g., soil type, topography), and anthropogenic factors (e.g., land-use change, international trade) to improve model accuracy. By expanding the range of contributing factors, the model can more comprehensively capture the diverse elements influencing species distribution, thereby providing more precise and reliable scientific support for predicting species’ habitat suitability and biodiversity conservation.

### 4.2. Invasion Risks and Control Strategies for S. scolytus Under Potential Distribution in China

*S. scolytus* is a key vector of Dutch elm disease (DED). When feeding at the leaf axils or branch forks of healthy elm trees, this beetle transmits fungal spores into the xylem. The pathogens then move within the plant to the leaves and roots, ultimately causing tree wilting and death [43,44,45]. Jürisoo et al. examined six bark beetle species, including *S. scolytus* and *S. triarmatus*, to compare the diversity and frequency of the Dutch elm disease (DED) pathogens they carried [43]. Among the fungal species detected, *Ophiostoma novo-ulmi* was the most prevalent, accounting for 8.2% of all isolates. Furthermore, among beetles carrying this pathogen, *S. scolytus* represented the highest proportions, exceeding one quarter of the infected individuals. In addition to fungal spores, *S. scolytus* frequently harbors symbiotic mites, including *Elattoma fraxini*, *Proctolaelaps scolyti*, and *Tarsonemus crassus*. These mites can carry DED pathogen spores on their body surface, within specialized structures such as sporothecae, or even inside their digestive tracts. It has been hypothesized that *P. scolyti* and *T. crassus* may increase the spore load on *S. scolytus*, thereby facilitating the infection of host plants by *O. novo-ulmi*. This mechanism may help explain the high efficiency of *S. scolytus* in transmitting DED [46]. In Russia, Ryss et al. reported that *S. scolytus* can carry a newly described nematode species, *Bursaphelenchus ulmophilus* sp. n., which appeared to be associated with symptoms of DED [47]. The authors hypothesized that synergistic interactions might exist between DED pathogens and *Bursaphelenchus* nematodes, potentially contributing to the development of disease in woody plants. Therefore, given the critical role of *S. scolytus* in DED transmission, determining its potentially suitable habitats is both a prerequisite and a theoretical basis for developing effective control measures.

In China, despite its limited potential distribution under near current climatic conditions, the suitable range is expected to expand under future climatic scenarios, even appearing moderately and highly suitable areas. Due to the beetle’s limited natural dispersal capacity, long-distance spread is predominantly facilitated by cross-border trade and transport activities. Provinces such as Jiangsu, Hunan, and Hubei possess ecological conditions conducive to the colonization of *S. scolytus*. Therefore, particular attention should be paid to the risk of transboundary introduction via commodity trade and cargo transport. Strengthening entry quarantine and implementing targeted preventive measures is essential to reduce the potential ecological threat posed by this species to China’s elm ecosystems.

Based on the current distribution range and host specificity of *S. scolytus*, quarantine efforts should be prioritized for elm timber and related products imported from high-risk countries and regions, including some Europe countries. Particular attention should be directed toward high-risk carriers such as minimally processed wood, raw logs, wooden packaging materials, bark, seedlings, and potted plants. For these commodities, raising quarantine standards and reinforcing fumigation and other phytosanitary treatments are advisable to further reduce the risk of pest introduction.

Additionally, inspection protocols should be strengthened for wood-based by-products such as wood chips, wood shavings, and discarded timber, which may act as passive vectors of the pest. Preventing their entry into China through international trade is critical for safeguarding domestic forest ecosystems.

As part of targeted prevention strategies, surveillance efforts should be particularly intensified at key coastal ports, including those in Jiangsu, Shanghai, and Zhejiang. Quarantine frequency for commodities associated with *S. scolytus* hosts should be increased, accompanied by improvements in diagnostic capacity and the refinement of quarantine identification protocols. These measures are essential to ensure that the customs inspection barrier remains stringent and effective. Inland provinces with predicted high suitability, such as Hunan, should establish early-warning systems and contingency plans to reduce the risk of post-introduction spread.

Through the implementation of these integrated measures, the invasion potential of *S. scolytus* can be effectively curtailed. These actions will contribute to the protection of China’s elm resources, preserve forest ecosystem stability, and strengthen national ecological security.

### 4.3. Impact of Global Climate Change on the Habitat Suitability of S. scolytus and Its Key Environmental Drivers

Under current climatic conditions, highly suitable areas for *S. scolytus* are mainly distributed across Europe and the eastern part of North America. Additionally, areas of medium to high suitability are also observed in the southwestern regions of South America and Australia. Under future climatic conditions, particularly under the SSP370 and SSP585 scenarios, environmental conditions in provinces such as Guangdong and Guizhou in China are projected to become increasingly favorable for the establishment of *S. scolytus*. Moreover, medium-to-high suitability zones are expected to emerge across several provinces, including Hunan, Jiangsu, and Zhejiang. These projections suggest that climate change is creating favorable opportunities for the colonization of *S. scolytus* in China. As a result, both the invasion risk and potential impacts are expected to increase substantially. Temperature and precipitation are critical in regulating species growth, reproduction, and population spread. Current findings indicate that the potential geographical distribution of *S. scolytus* is mainly determined by key environmental variables, such as the minimum temperature of the coldest month, precipitation seasonality, and the precipitation of the driest quarter. In regions identified as climatically suitable for *S. scolytus*, heightened attention should be given to the potential for invasion, with prevention and control strategies promptly adjusted according to the actual implementation and effectiveness of quarantine measures.

Organisms with comparable ecological requirements often exhibit similar patterns in the prediction of potential suitable areas. For example, Zhou Yuting et al. identified precipitation in the driest season and the minimum temperature of the coldest month as the most influential bioclimatic factors when modeling the global and Chinese habitat suitability of *Hylurgus ligniperda* [48]. This species showed high adaptability in the coastal and adjacent regions of Shandong and Jiangsu provinces in China. Similarly, Huang Dao et al. predicted the distribution of *Xyleborinus saxesenii* and reported that its suitable habitats were mainly located in Europe, the mountainous regions of western North America, the eastern and southeastern coasts of South America, and eastern Asia. Future projections indicated a potential expansion into northwestern China, likely driven by global warming and its effect on latitudinal and elevational range shifts [49].

Furthermore, studies predicting suitable breeding areas consistently emphasize the critical roles of temperature and precipitation. Global warming is expected to alter these parameters, thereby influencing pest distribution patterns. For instance, Bentz et al. examined the climatic adaptability of *Dendroctonus ponderosae* and *D. rufipennis*, revealing that rising temperatures may enhance growth rates and overwintering survival, ultimately promoting range expansion toward higher latitudes and elevations [50]. Their simulation results indicated an increased outbreak potential for both species throughout the 21st century, posing a significant threat to forest ecosystems. Similarly, Antonio González-Hernández et al. identified annual temperature variability, the mean temperature of the wettest quarter, and the mean temperature of the warmest quarter as the main contributors to the distribution of *Dendroctonus mexicanus* [51]. At present, this species is predominantly found in the Trans-Mexican Volcanic Belt and the Sierra Madre Occidental, but future warming may drive its expansion into higher elevations and northern regions. Zheng Xiaoyi et al. reported that suitable areas for *Euwallacea fornicatus* were strongly associated with the mean temperature of the coldest season, precipitation during the wettest season, and precipitation in the coldest season [52]. Currently, its core suitable habitats are concentrated in Guizhou Province and adjacent areas. Future climate warming may facilitate a shift toward high-latitude and high-altitude zones such as Chongqing and Sichuan. In addition, Zhou Yuting et al. investigated the global suitability of *Dendroctonus valens* and its symbiotic fungus *Leptographium procerum* [53]. The beetle’s suitable range was mainly located in central and southern North America, as well as central and northeastern China, whereas the fungus was predicted to thrive in eastern North America, the Andes, and northern China. Under future climate scenarios, both species are expected to shift their ranges northward, reflecting a coordinated response to warming temperatures.

Under future climate scenarios, the highly suitable habitat of *S. scolytus* is projected to expand from eastern coastal cities, such as Shanghai and Zhejiang, toward inland regions, particularly provinces such as Hunan. As global climate change intensifies, rising temperatures and altered precipitation regimes in inland areas may create favorable environmental conditions for the species’ survival and reproduction.

This trend suggests that climate warming could accelerate the inland expansion of suitable habitats, thereby increasing the potential invasion risk. Therefore, enhanced surveillance and early-warning systems are urgently required in vulnerable inland regions. Particular attention should be directed toward areas where habitat suitability is expected to increase. In these zones, targeted management strategies must be developed and implemented to mitigate the potential impacts on elm resources and associated ecosystems in China.

## 5. Conclusions

Based on the occurrence points and key environmental variables of *S. scolytus*, we selected nine models (GLM, GAM, CTA, ANN, SRE, FDA, MARS, RF, and MaxEnt) to establish an ensemble model to predict its potential distribution under near current and future conditions (SSP370, SSP585). The results revealed that, at a global scale, the potential geographical distribution for *S. scolytus* were primarily distributed across temperate zones between 30° N and 60° N, including Europe, eastern Asia, eastern and northwestern North America, and parts of southern and northeastern South America. In the case of China, under near-current climatic conditions, the suitable area of *S. scolytus* was relatively limited and mainly distributed in provinces such as Hunan, Hubei, Zhejiang, and Fujian. Under the SSP370 and SSP585 scenarios, the potential suitable area is projected to gradually expand towards the southwest and south, with moderately or highly suitable habitats even appearing in regions such as Hunan and Zhejiang. *S. scolytus* is an important carrier of DED. Therefore, in regions with a high risk of colonization, strict inspections should be conducted on elm logs, seedlings, and wooden packaging materials originating from areas where *S. scolytus* and DED occur. Relevant quarantine treatment certificates should be verified to cut off the pathways of invasive risks from outside.

## Figures and Tables

**Table 1:** Selection of model algorithm.

Model	Overview	Biomod2 Dependent on Packages
MaxEnt	Uses the maximum entropy principle to model the conditional probability distribution, often used in classification and NLP tasks. Typically optimized using iterative methods like gradient ascent [30].	maxent, dismo
GLM	Fits quadratic response curves with no interactions between covariates, using stepwise backward selection [15].	glm
GAM	Fits smoothed additive response curves through the mgcv package [31].	gam, mgcv
MARS	Fits complex response curves by joining linear segments, using the earth package [32].	earth
ANN	A neural network with one hidden layer optimized using cross validation [33].	nnet
CTA	Decision tree analysis with complex trees, using the rpart package [34].	rpart
FDA	Uses MARS for dimensionality reduction before classification, fitted through mda [35].	mda
RF	Ensembles predictions from 500 random forest trees, using randomForest package [36].	randomForest
SRE	A data dimensionality reduction algorithm applicable to nonlinear data [37].	gbm

**Table 2:** The importance of selected environmental factors.

Environmental Factors	Average Importance Value	Average Importance Percentage of Total Importance
bio6	0.1460	0.3570
bio15	0.1140	0.2790
bio17	0.0444	0.1080
bio3	0.0295	0.0722
bio8	0.0254	0.0622
bio16	0.0231	0.0564
bio18	0.0212	0.0518
bio2	0.0053	0.0129

## Data Availability

The original contributions presented in this study are included in the article. Further inquiries can be directed to the corresponding authors.

## Abbreviations

The following abbreviations are used in this manuscript:

SDMs	Suitable distribution models
BCC-CSM2-MR	Beijing Climate Center climate system model version 2—medium resolution
SSP	Shared socioeconomic pathway
MaxEnt	Maximum entropy
GLM	Generalized linear model
GAM	Generalized additive model
MARS	Multivariate adaptive regression splines
ANN	Artificial neural networks
CTA	Classification tree analysis
FDA	Flexible discriminant analysis
RF	Random forest
SRE	Spectral Relaxation Embedding
ROC	Receiver operating characteristic
TPR	True-positive rate
FPR	False-positive rate
AUC	Area under the ROC curve
TSS	True skill statistic
